# Impact of Reference Gene Selection for Target Gene Normalization on Experimental Outcome Using Real-Time qRT-PCR in Adipocytes

**DOI:** 10.1371/journal.pone.0015208

**Published:** 2010-12-13

**Authors:** Bradley S. Ferguson, Heesun Nam, Robin G. Hopkins, Ron F. Morrison

**Affiliations:** Department of Nutrition, The University of North Carolina at Greensboro, Greensboro, North Carolina, United States of America; Brigham and Women's Hospital, United States of America

## Abstract

**Background:**

With the current rise in obesity-related morbidities, real-time quantitative reverse transcription polymerase chain reaction (qRT-PCR) has become a widely used method for assessment of genes expressed and regulated by adipocytes. In order to measure accurate changes in relative gene expression and monitor intersample variability, normalization to endogenous control genes that do not change in relative expression is commonly used with qRT-PCR determinations. However, historical evidence has clearly demonstrated that the expression profiles of traditional control genes (e.g., β-actin, GAPDH, α-tubulin) are differentially regulated across multiple tissue types and experimental conditions.

**Methodology/Principal Findings:**

Therefore, we validated six commonly used endogenous control genes under diverse experimental conditions of inflammatory stress, oxidative stress, synchronous cell cycle progression and cellular differentiation in 3T3-L1 adipocytes using TaqMan qRT-PCR. Under each study condition, we further evaluated the impact of reference gene selection on experimental outcome using examples of target genes relevant to adipocyte function and differentiation. We demonstrate that multiple reference genes are regulated in a condition-specific manner that is not suitable for use in target gene normalization.

**Conclusion/Significance:**

Data are presented demonstrating that inappropriate reference gene selection can have profound influence on study conclusions ranging from divergent statistical outcome to inaccurate data interpretation of significant magnitude. This study validated the use of endogenous controls in 3T3-L1 adipocytes and highlights the impact of inappropriate reference gene selection on data interpretation and study conclusions.

## Introduction

The obesity epidemic has led to a plethora of investigations examining mechanisms that regulate adipocyte differentiation and function as well as the role adipose tissue plays in the development of insulin resistance, diabetes and heart disease. As our understanding of the adipocyte has progressed from that of a storage depot to an endocrine cell, there is increased need to examine relative expression of low-abundance genes (e.g., cytokines, adipokines) involved in metabolic regulation from a tissue that traditionally yields limited RNA [1]–[4]. While earlier work with conventional methodology provided qualitative assessment of mRNA abundance, the quantitative nature of real time qRT-PCR affords a measure of sensitivity that is suited for reliable detection of 2-fold changes in gene expression over dynamic ranges of starting material [2], [5]. This methodology comes with a price, however, as increased sensitivity of qRT-PCR along with inherent variability in biological systems, experimental and extraction protocol disparity, as well as differences in reverse transcription and PCR efficiencies makes normalization of real-time data an absolute requirement for accurate data interpretation regarding genes of interest [5]–[8].

Several strategies have been proposed for normalization of qRT-PCR data, the most common of which involves analysis of a co-expressed endogenous control (i.e., reference gene) whose relative expression should not change with treatment or study conditions [5], [7], [9]. When these criteria are strictly met, this strategy would be expected to ‘normalize’ confounding variation due to intersample variability such as differences in PCR efficiency or loading disparity. β-actin, glyceraldehyde 3-phosphate dehydrogenase (GAPDH) and α-tubulin have been traditionally used as control, ‘house-keeping’ genes for Northern blotting and other conventional, less sensitive assays, in spite of decades of reports clearly demonstrating expression profiles that vary markedly based on cellular phenotype and experimental design [10]–[12]. While some reports contend that overall study conclusions would remain the same as the variability in a reference gene would be similar between study and control groups [13], others note that normalization to an inappropriate endogenous control may lead to misinterpretation and confounding data with qRT-PCR [7], [14], [15]. While numerous reports have evaluated changes in reference gene expression under various experimental conditions [2], [4], [15], few have explored the impact of reference gene selection on data interpretation and study conclusions [14].

To evaluate the impact of reference gene selection on experimental outcome, we validated six commonly used reference genes including GAPDH, β-actin, transferrin receptor (TfR), cyclophilin A (cyc), α-tubulin (α-tub) and 18 ribosomal RNA (18S) using TaqMan qRT-PCR chemistry and methodology in the well-established 3T3-L1 adipocyte cell line under four diverse study conditions including inflammatory stress, oxidative stress, synchronous cell cycle progression and cellular differentiation. Under each study condition, data are presented demonstrating the impact of reference gene selection on normalized target gene expression. This report clearly demonstrates the critical importance of reference gene validation for all experimental conditions regarding significance, interpretation, and experimental outcome when using the sensitivity and reliability of qRT-PCR for assessment of relative gene expression.

## Results

### Effect of small variation in reference gene expression on statistical significance

A widely used method of correcting for intersample variability using qRT-PCR involves normalizing to one or more reference genes whose expression should not change with treatment or between study conditions [5], [7], [9]. Thus, it is important to distinguish technical variability from true biological changes in gene expression. For this purpose, we chose to use guidelines previously described by Gorzelniak et al [2], whereby reference genes are classified based on treated and untreated differences in cycle number when an amplified probe crosses the threshold (ΔC_T_) during qRT-PCR reactions. According to these guidelines, ΔC_T_ values ≤ +/−0.5 are considered fluctuation in gene expression that is largely due to technical variance (e.g., unequal loading, PCR efficiency, etc.) that should be reflected similarly between both reference and target genes. Conversely, ΔC_T_ values ≥ +/−1.0 are strongly suggestive of biological variability resulting from treatment or study conditions, precluding the use of such reference genes for target gene normalization. To gain insight into ΔC_T_ values that fall between 0.5 and 1.0, we extended the guideline by observing reference gene expression over time following treatment for consistent direction shifts between contiguous data points that would be suggestive of biological variance.

**Figure 1:**
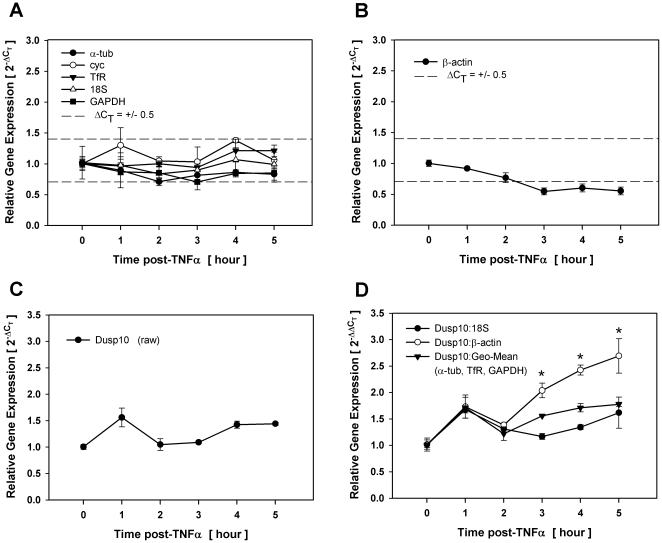
Gain of statistical significance with inappropriate reference gene selection. Total RNA was isolated over time from density-arrested 3T3-L1 preadipocytes treated with 1 nM TNFα and assessed for reference and target gene expression by qRT-PCR. Fold changes in reference genes that fell within (A) and outside (B) the ΔC_T_ ≤ +/−0.5 limits of suitability. Fold changes in Dusp10 gene expression without (C) and with normalization (D) to 18S, the geometric mean of three reference genes or β-actin. Asterisks indicates significant differences between treated and untreated samples (p<0.05).

Using the criterion of ΔC_T_ ≤ +/−0.5 as a delimiter of reference gene suitability, we evaluated six commonly used reference genes (Table 1) over time in undifferentiated 3T3-L1 preadipocytes following exposure to either 1 nM TNFα or 300 µM H_2_O_2_, representing conditions of inflammatory and oxidative stress, respectively. As ΔC_T_ values are exponents and should not be statistically evaluated [16], all validation data were converted to ‘fold-changes’ using the 2^−ΔC^_T_ method for raw data or 2^−ΔΔC^_T_ method for normalized data. Accordingly, ΔC_T_ of +0.5 and −0.5 is equivalent to 0.7 and 1.4 fold changes in relative gene expression, respectively. As illustrated in Fig. 1A, relative expression of most (5 of 6) reference genes tested (α-tub, cyc, TfR, GAPDH and 18S) fluctuated within the ΔC_T_ ≤ +/−0.5 limits in a manner that would be consistent with intersample variability and, thus, considered suitable for use in target gene normalization following TNFα exposure. In contrast,β-actin steadily and consistently dropped below the ΔC_T_ ≤ +/−0.5 at all time points following 3 hrs post-TNFα stimulation (Fig. 1B). As the ΔC_T_ for β-actin closely approximated a value of 1.0 (i.e., 0.5-fold decrease) at contiguous time points, these changes were likely due to biological variability, thus precluding this commonly used reference gene for suitability under these experimental conditions.

**Table 1:** TaqMan primer probes used for this study.

symbol	gene name	accession	manufacturer's no	length	C_T_
	*reference genes*				
Actb	β-actin	NM_007393.3	Mm00607939_s1	115	17
Gapdh	glyceraldehyde-3-phosphate dehydrogenase (GAPDH)	NM_008084.2	Mm99999915_g1	107	17
Tuba1a	α-tubulin (α-tub)	NM_011653.2	Mm00846967_g1	123	23
Ppia	peptidylprolyl isomerase A (cyclophilin A (cyc))	NM_008907.1	Mm02342430_g1	148	17
Tfrc	transferrin receptor (TfR)	NM_011638.3	Mm00441941_m1	66	26
18S	18 ribosomal RNA	X03205.1	4342930E	187	9
	*target genes*				
Dusp3	dual specificity phosphatase-3	NM_028207.2	Mm00459216_m1	59	26
Dusp10	dual specificity phosphatase-10	NM_022019.5	Mm00517678_m1	104	26
Cebpb	CAATT/enhancer binding protein beta (C/EBPβ)	NM_009883.3	Mm00843434_s1	159	25
Ccna2	cyclin A2	NM_009828.2	Mm00438064_m1	90	23
Pparg	peroxisome proliferator activator receptor γ (PPARγ)	NM_001127330.1	Mm00440945_m1	105	25

To assess the impact of using β-actin as a reference gene, we examined relative expression of dual specificity phosphatase-10 (Dusp10) without and with normalization to either 18S, the geometric mean of three stable reference genes (α-tub, TfR, GAPDH) or β-actin following TNFα exposure. Dusp10 was chosen for this experiment as a gene that is not responsive to TNFα stimulation under these conditions. As illustrated in Fig. 1D, fold changes in Dusp10 gene expression when normalized to 18S or the geometric mean of three reference genes closely approximated fold changes as shown for raw Dusp10 without normalization (Fig. 1C), where data points following treatment were not significantly different from untreated controls. In contrast, when Dusp10 was normalized to β-actin, each point where β-actin fell outside the ΔC_T_ ≤ +/−0.5 delimiter range was significantly different from untreated controls. Thus, normalizing Dusp10 to β-actin resulted in an inaccurate ‘gain’ of significance that did not occur with 18S or the geometric mean of stable reference genes.

**Figure 2:**
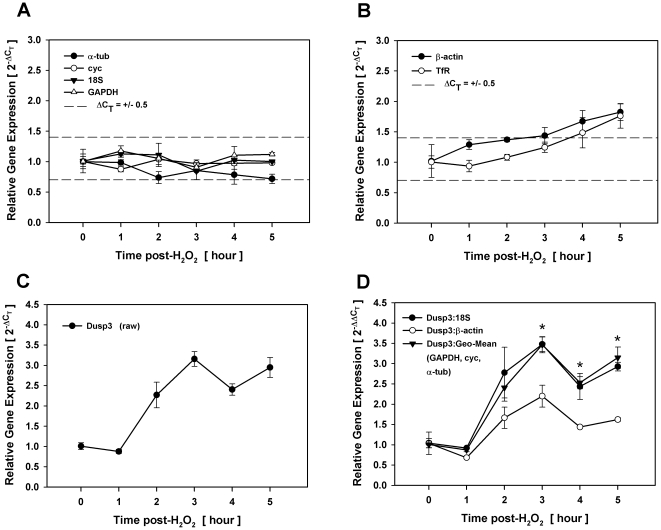
Loss of statistical significance with inappropriate reference gene selection. Total RNA was isolated over time from density-arrested 3T3-L1 preadipocytes treated with 300 µM H_2_O_2_ and assessed for reference and target gene expression by qRT-PCR. Fold changes in reference genes that fell within (A) and outside (B) the ΔC_T_ ≤ +/−0.5 limits of suitability. Fold changes in Dusp3 gene expression without (C) and with normalization (D) to 18S, the geometric mean of three reference genes, or β-actin. Asterisks indicates significant differences between treated and untreated samples (p<0.05).

Similar implications regarding inappropriate reference gene selection were observed under conditions of oxidative stress. As shown in Fig. 2B, β-actin and TfR were the only two reference genes that increased steadily above ΔC_T_ ≤ +/−0.5 range at more than one contiguous time point. To assess the impact of reference gene selection, we examined relative changes in Dusp3 gene expression without and with normalization to either 18S, the geometric mean of three reference genes (GAPDH, cyc, α-tub) or β-actin following H_2_O_2_ exposure. Dusp3 was chosen for this experiment as a gene that displays modest (∼3 fold), but significant increases in response to H_2_O_2_ stimulation. When Dusp3 was normalized to 18S or the geometric mean of stable reference genes (Fig. 2D), fold changes closely approximated those shown for raw Dusp3 without normalization (Fig. 2C), where gene expression was significantly different from untreated controls at each point following 3 hrs post-H_2_O_2_ exposure. When Dusp3 was normalized to β-actin, however, the target gene was no longer significantly different from untreated controls at any point where this reference gene fell outside the ΔC_T_ ≤ +/−0.5 range of suitability. Thus, normalizing Dusp3 to β-actin under conditions of oxidative stress resulted in an inaccurate ‘loss’ of significance that did not occur with 18S or the geometric mean of stable reference genes. Collectively, these data demonstrate that statistical significance is highly dependent on appropriate reference gene selection, even when changes in reference gene expression are marginal.

### Effect of large variation in reference gene expression on experimental outcome

**Figure 3:**
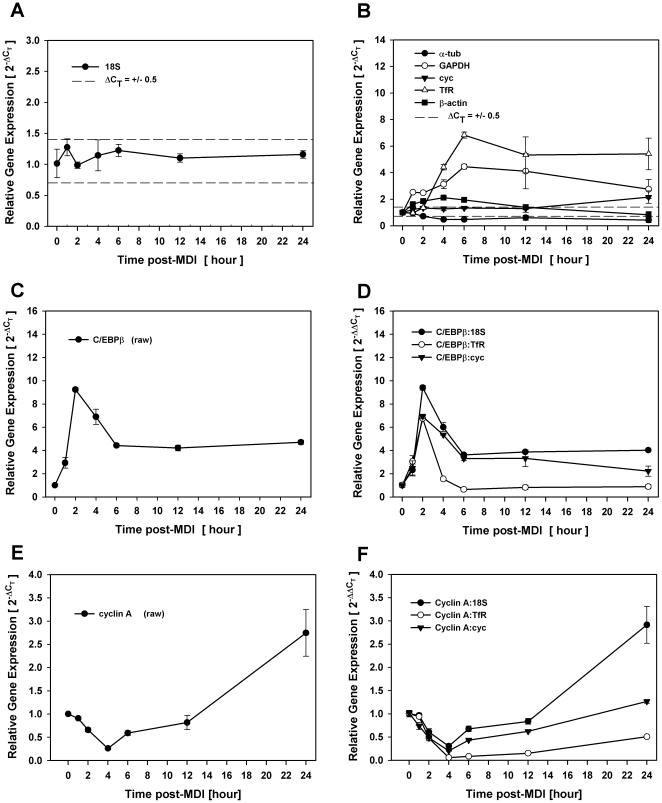
Inaccurate experimental outcome with inappropriate reference gene selection. Total RNA was isolated over time (0–24 hrs) from density-arrested 3T3-L1 preadipocytes treated with MDI and assessed for reference and target gene expression by qRT-PCR during early stages of differentiation. Fold changes in reference genes that fell within (A) and outside (B) the ΔC_T_ ≤ +/−0.5 limits of suitability. Fold changes in C/EBPβ gene expression without (C) and with normalization (D) to 18S, cyc, or TfR. Fold changes in cyclin A gene expression without (E) and with normalization (F) to 18S, cyc, or TfR.

To examine the impact of considerable reference gene variability on target gene expression, we validated the same six reference genes during diverse experimental conditions of proliferation and differentiation, both of which have been known for decades to markedly alter the expression of commonly used reference genes [11], [12]. Early stages of 3T3-L1 adipocyte differentiation are characterized by synchronous cell cycle progression [17], [18], where quiescent preadipocytes cycle through one complete round of cell division within 24 hrs post-MDI concurrent with the onset of early stages of adipocyte gene expression [19], [20]. As shown in Fig. 3A, 18S was the only reference control that did not fluctuate beyond the ΔC_T_ ≤ +/−0.5 range of suitability at any time point during the first 24 hrs of adipocyte differentiation. In contrast, most (4 of 6) of the reference genes were disqualified as appropriate reference controls based on fold changes that exceeded this range at more than one contiguous time point with GAPDH and TfR displaying marked increases (>4-fold) clearly indicative of biological variability. Cyclophilin A expression fell outside the delimiter range only at the 24 hr time point with a 2-fold increase in gene expression (Fig. 3B).

To assess the impact of reference gene selection under these conditions, we evaluated two unrelated target genes with well-established roles during this early phase of adipocyte differentiation. First, we examined the expression profile of the transcription factor, C/EBPβ, which is known for its role in initiating a cascade of transcriptional events that culminate in acquisition of the mature adipocyte phenotype [21]–[25]. Normalization of C/EBPβ to 18S resulted in fold changes in gene expression (Fig. 3D) that were nearly identical to fold changes in raw expression without normalization (Fig. 3C) where 2 hrs post-MDI yielded 9-fold induction in target gene expression that decreased to 4-fold induction by 6 hrs where it remained consistently and steadily elevated for 24 hrs. These results were very similar to the well-documented expression profile of C/EBPβ using conventional methodology and consistent with its role in regulating the expression of C/EBPα and PPARγ, two master regulators of adipogenesis that are induced ∼48 hrs into differentiation [25], [26]. In contrast, however, the prolonged increase in gene expression beyond 6 hrs post-MDI was not evident when C/EBPβ was normalized to TfR, where the 6-fold induction of the reference gene ablated the 4-fold induction of the target gene. As our screen did not identify three stable reference genes that could be grouped in a geometric mean, we further normalized C/EBPβ to cyclophilin A that fell outside the ΔC_T_ ≤ +/−0.5 range of suitability only at the 24 hr time point. As shown in Fig. 3D, C/EBPβ normalized to cyclophilin A resulted in fold changes that closely approximated both raw C/EBPβ as well as that normalized to 18S at all time points where this reference gene fell within the ΔC_T_ ≤ +/−0.5 limits. However, the two-fold increase in cyclophilin at 24 hrs post-MDI resulted in a 50% reduction of target gene expression at that time point.

We next evaluated the expression profile of cyclin A, which is well known for its regulatory role during S phase of proliferation [27], [28] where mRNA has been shown to be elevated between 16 hrs and 30 hrs post-MDI [26]. Similarly, the expression profile was nearly identical when comparing raw cyclin A expression (Fig. 3E) to that which was normalized to 18S (Fig. 3F), where expression initially decreased below baseline levels before increasing to a 3-fold induction by 24 hrs. When the same target gene was normalized to TfR instead, the >4-fold increase in TfR at 6 hrs post-MDI resulted in a dramatic drop in cyclin A expression that never increased above untreated basal levels throughout the course of cell cycle progression (Fig. 3F). We also normalized cyclin A to cyclophilin A as discussed above where the expression profile approximated that observed for raw cyclin A as well as cyclin A normalized to 18S, at all time points where cyclophilin fluctuated within the ΔC_T_ ≤ +/−0.5 range of suitability. However, at the one time point where cyclophilin exceeded that range, the 3-fold increase in cyclin A was erroneously ablated by the 2-fold increase in cyclophilin expression.

**Figure 4:**
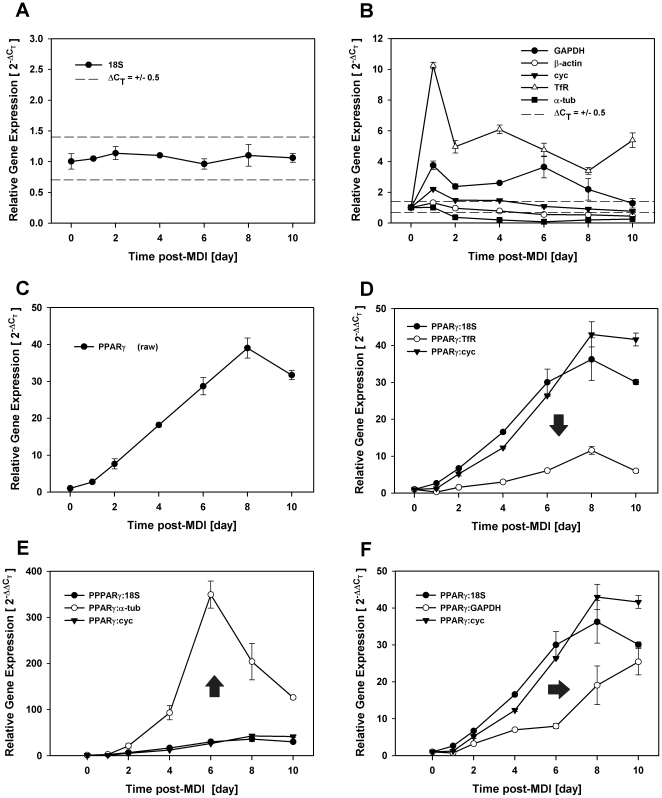
Directional shifts in experimental outcome with inappropriate reference gene selection. Total RNA was isolated over time (0–10 days) from density-arrested 3T3-L1 preadipocytes treated with MDI and assessed for reference and target gene expression by qRT-PCR during complete adipocyte differentiation. Fold changes in reference genes that fell within (A) and outside (B) the ΔC_T_ ≤ +/−0.5 limits of suitability. Fold changes in PPARγ gene expression without (C) and with normalization to 18S, cyc, or TfR (D), 18S, cyc, or α-tubulin (E), and 18S, cyc, or GAPDH (F). Arrows indicate directional shifts relative to normalization to 18S.

We further evaluated reference gene selection over the entire course of differentiation (0–10 days post-MDI) where 18S was again the only reference control examined that fluctuated clearly within our ΔC_T_ ≤ +/−0.5 range of suitability (Fig. 4A). Under these conditions, most (4 of 6) reference genes tested were disqualified as suitable reference genes as expression exceeded the delimiter range at more than one contiguous time point with TfR and GAPDH displaying marked increases and α-tubulin marked decreases in relative expression clearly indicative of biological variability. Cyclophilin A fluctuated beyond the ΔC_T_ ≤ +/−0.5 range only at 24 hrs post-MDI. To illustrate the impact of reference gene selection under these conditions, we examined the expression profile of a single target gene individually normalized to five different reference genes of varying degrees of suitability. For this study, we chose the transcription factor, PPARγ, whose function is well-established as a dominant transcriptional regulator of adipogenesis [29]–[31]. As expected, expression profiles were nearly identical, by kinetics and magnitude, when comparing raw PPARγ expression (Fig. 4C) to that which was normalized to 18S (Fig. 4D), where expression gradually increased from 2 days post-MDI reaching a plateau at 8 days post-MDI with a 40-fold induction in gene expression. As cyclophilin A fell outside the ΔC_T_ ≤ +/−0.5 range only at one time point prior to the induction of PPARγ, we noted little deviation in magnitude or kinetics when comparing PPARγ normalized to cyclophilin A versus 18S. In contrast, the magnitude of PPARγ expression was dramatically attenuated when normalized to TfR as compared to 18S reaching only a 10-fold increase in gene expression (Fig. 4D). Conversely, the magnitude of PPARγ expression was dramatically increased when normalized to α-tubulin reaching nearly a 350-fold increase in gene expression at 6 days post-MDI (Fig. 4E). Although end point magnitude was nearly identical, normalizing PPARγ to GAPDH resulted in a dramatic delay in the kinetics of PPARγ mRNA accumulation as compared to 18S (Fig. 4F). Collectively, these data demonstrate that reference gene selection can have a profound impact on experimental outcome illustrating the critical need to validate each reference gene in a cell-type and condition-specific manner.

## Discussion

Our report presents empirical evidence demonstrating that inappropriate reference gene selection for qRT-PCR normalization can have profound influence on study conclusions ranging from divergent statistical outcome to inaccurate data interpretation of significant magnitude. First, we demonstrate that many commonly used reference genes fluctuate in a condition-specific manner beyond the limits of suitability when normalizing for target gene expression using qRT-PCR. Second, we illustrate that even small variability in reference gene expression can have a marked influence on the presence or absence of statistical significance between study groups, especially when target genes show modest changes in mRNA abundance. Third, this report demonstrates that improper selection of a suitable reference gene can lead to grossly inaccurate data that would significantly affect interpretation of biological outcome and study conclusions. These data collectively demonstrate that validation of each reference gene on an individual basis for all treatment and experimental conditions is paramount for accurate data interpretation when normalizing for intersample variability involving qRT-PCR.

Data presented here and elsewhere [14] clearly demonstrate that whether statistically significant differences between study groups are present or absent can depend on which reference gene is used for normalization even when variability in reference gene expression is marginal. Comparing the outcome of normalizing to β-actin versus 18S, we present data from two genes; Dusp10 whose raw profile did not vary over time following TNFα (Fig. 1) and Dusp3 that increased following H_2_O_2_ with statistical significance. Neither the expression profile nor significance in gene expression of either gene was markedly influenced by normalizing to 18S or the geometric mean of three stable reference genes. In contrast, normalizing Dusp10 and Dusp3 to β-actin resulted in an inaccurate gain or loss of significance, respectively, demonstrating that even small, less than 2-fold variation in reference gene expression can have a significant influence on statistical outcome. Both experiments presented evidence of biological variability as β-actin expression approached 2-fold differences between treated and untreated conditions at contiguous time points following stimulation strongly implicating the effect of experimental treatment on relative gene expression. It is also interesting to note that these changes in reference gene expression had a discernible impact on significance at all time points where β-actin exceeded the ΔC_T_ ≤ +/−0.5 range of suitability. Whether or not the variability of β-actin under these experimental conditions was representative of true biological change in gene expression can be argued, these data clearly demonstrate that careful reference gene validation is critical when considering statistical significance of target genes, especially those that present with marginal changes in gene expression.

This investigation further demonstrates that grossly inaccurate conclusions can result from studies where reference genes show marked differences in mRNA abundance that clearly result from treatment or study conditions. Comparing the outcome of normalizing to a variety of conventionally used reference genes that presented with indisputable biological variability, we presented data from two experiments involving three target genes with well-defined roles in adipocyte differentiation where reference gene selection had an unequivocal impact on data interpretation. The first of these experiments (Fig. 3) examined the expression profile of C/EBPβ and cyclin A during the first 24 hrs of differentiation, which is known to include a phase of synchronous cell cycle progression. While the expression profile of either gene was not markedly influenced when normalizing to 18S, the well-documented prolonged expression of C/EBPβ during this time period as well as the induction of cyclin A at 24 hrs required for S phase progression was completely ablated when normalizing to TfR that presented with its own 6-fold increase in gene expression. Simply normalizing to this conventionally used reference gene without validation would have resulted in grossly inaccurate data as the outcome would have been that the expression of both genes decreased, rather than increased, relative to unstimulated basal expression at 24 hrs of differentiation. Similar impact on experimental outcome was also noted in the second experiment (Fig. 4) where PPARγ expression was individually normalized to a variety of reference genes over the entire course of differentiation. While the expression profile was not markedly influenced when normalized to 18S, the well-documented induction of this target gene was markedly suppressed when normalized to TfR, increased when normalized to α-tub, and delayed when normalized to GAPDH. In this example, normalizing to inappropriate reference genes markedly altered both kinetics and magnitude of target gene expression to an extent that would unquestionably lead to erroneous data interpretation and study conclusions.

Based on the unequivocal impact of reference gene selection on experimental outcome presented here and elsewhere [14], there is a clear need for guidelines concerning tolerable variability of reference genes used for data normalization [32]. In this report, we chose a criterion defined by others [2] that quantifies changes in C_T_ values of a given reference gene between treated and untreated conditions. We extended the original criterion by examining changes in C_T_ values over time following treatment so as to determine whether modest fluctuations presented with sporadic directional swings consistent with ‘noise’ as opposed to trends in contiguous data points consistent with biological effects on gene expression. This extension was considered particularly valuable when evaluating changes in C_T_ values between 0.5 and 1.0 as shown here for β-actin where the biological trend in reference gene expression resulted in the erroneous gain or loss of statistical significance regarding genes of interest. As qRT-PCR protocols have been refined, it has become well-established that changes in C_T_ values ≥ 1.0, representing ≥ 2-fold changes in gene expression are most likely due to biological variance [2] and ABI (P/N 4371001 Rev A). Thus, reference genes that present with ΔC_T_ values ≥ 1.0 should not be used for normalization, if indeed the observed changes in gene expression are due to biological versus technical variance, which may be difficult to discern in some circumstances. Arguably, use of reference genes that display 2-fold changes in gene expression could serve the purpose of normalization, but only if the variance is technical in nature requiring additional measures (e.g., evaluation over time, etc) to establish suitability when using a single reference gene.

To address these concerns, others have proposed normalizing target gene expression to the geometric mean of three stable reference genes [33]. While equivalent directional shifts among three reference genes would be strongly indicative of technical variation supporting the use of ΔC_T_ values exceeding 1.0, this method of normalization, which would be reagent and labor expensive, would still require validation to identify three references genes that do not display indices of biological variance as marked deviation of even a single gene could significantly affect the geometric mean of multiple genes resulting in erroneous data interpretation. In some circumstances, the added expense may not be necessary as shown here and by others [34], where experimental outcome using a single, stable reference gene was equivalent to that observed when using the geometric mean of multiple, stable reference genes. It may also be necessary to validate more than three reference genes in order to identify at least three that are not affected by biological variance as shown in this report where validation of as many as six reference genes during adipocyte differentiation failed to identify three reference genes that displayed stable expression suitable for inclusion in geometric mean analyses. Therefore, it is imperative that all reference genes are validated for each experimental condition regardless of whether a single reference gene or the geometric mean of multiple reference genes is the chosen method of normalization. This premise should apply even when using established algorithms that quantify gene stability [4] as variation between tissue and cell type or experimental treatment may lead to biological changes in gene expression that is otherwise stable or at least unaffected by biological variance.

Albeit normalizing target gene expression to one or more reference genes is the method of choice of many investigators [5], [16], it is important to note that some situations may warrant the evaluation of raw target gene expression that is not corrected for technical variance. As reference gene validation presents with its own set of difficulties, others have also proposed normalizing target gene expression to total RNA [6], [35]. However, neither method corrects for technical variation imposed by pipetting error, differences in PCR efficiency, or reaction interference imposed by contaminating reagents such as salts and phenol. It remains a matter of debate as to which method results in the most accurate experimental outcome. As one size may not fit all, any given technique may represent the best approach for a given set of circumstances or experimental conditions leaving the investigator to evaluate each method as warranted by the specific experiment under investigation.

Validation criteria used in this report demonstrated that 18S rRNA presented with the least degree of fluctuation of the six endogenous controls tested with ΔC_T_ ≤ +/−0.5 under four diverse experimental conditions. While 18S has been reported as the most stable endogenous control under various experimental conditions [15], [36], including omental and subcutaneous adipose tissues from obese and diabetic patients [15], others have noted modest variability in 18S following hormonal challenge of cultured human adipocytes [2]. Furthermore, 18S has been reported as the most unstable of endogenous controls tested in human visceral adipose tissue using various algorithms that quantify gene stability [4]. Interestingly, the latter report also demonstrated β-actin and GAPDH as among the most stable of reference genes tested under their defined experimental conditions. Although validated by different criteria, we do not find these reports contradictory as the results are specific for the experimental conditions under investigation. More to our point, we feel the diversity of these reports suggest the critical importance of validating all reference genes under all experimental conditions by all investigators. Even in our report, conventionally used reference genes such as GAPDH, α-tub, and TfR that were suitable under one condition, were deemed highly inappropriate for another condition in the same cell type. The blind use of any reference gene, including 18S rRNA, simply because it is commonly reported in the literature should not be the reason for selection of any control when normalizing target gene expression as measured by qRT-PCR.

While our studies highlighted 18S as the most stable of endogenous controls under all four of our defined experimental conditions, it should be noted that there are concerns with using rRNA as a control which may not be influenced by degradation machinery in a matter similar to mRNA [15], [37]. Moreover, there are some [37] that question whether 18S represents the best reference control when normalizing a low abundance target gene that crosses the threshold at 24 cycles relative to the very abundant 18S with a C_T_ value of 9 cycles (Table 1), corresponding to a 32,000 fold-difference in gene expression. To address these concerns, we determined that 18S was linear from 50 ng to 5 pg in our TaqMan assay and that the slope was nearly identical to those from calibration curves generated for each target gene (data not shown). Furthermore, we observed no differences in relative target gene expression when normalized to undiluted 18S at 50ng compared to diluted 18S at 50 pg, representing a 10 cycle difference in C_T_ value. While these data support the use of 18S as a suitable control for normalization, it should not be inferred that it was the only valid control in our study, nor should it be used without validation under each experimental condition.

In summary, we validated six commonly used reference genes for the purpose of evaluating the impact of reference gene selection on normalization of five target genes under diverse experimental conditions relevant to adipocyte biology. Data are presented demonstrating that inappropriate reference gene selection can have profound influence on study conclusions ranging from divergent statistical outcome to inaccurate data interpretation of significant magnitude. As all reference genes will likely fluctuate to unacceptable degrees of variability in one condition or another, data presented here strongly caution against the blind use of any endogenous control that has not been validated specifically for the experimental conditions under study. Use of unvalidated controls has lead to flawed outcomes where reported changes in target gene expression were actually due to changes in reference gene expression [5], [7], [38]. Until reference genes are evaluated on an individual basis for all experimental conditions, the erroneous impact of inappropriate reference gene selection on data interpretation and biological outcome will undoubtedly continue to contribute to inaccurate study conclusions and inconsistencies between reports.

## Materials and Methods

### Cell Culture and Differentiation Protocol

Murine 3T3-L1 preadipocytes, obtained from Howard Green, Harvard Medical School [39], were induced to differentiate as previously described [17]. Briefly, cells were propagated in growth medium containing DMEM supplemented with 10% calf bovine serum until reaching density-arrest at 2 days post-confluence. For partial determinations, total RNA was harvested over time from density-arrested preadipocytes treated with 1 nM TNFα or 300 µM H_2_O_2_ representing conditions of inflammatory or oxidative stress, respectively. For other determinations, density-arrested preadipocytes were induced to differentiate with a hormonal cocktail containing DMEM supplemented 10% fetal bovine serum (FBS), 0.5 mM, 1-methyl-3-isobutylxanthine, 1 µM dexamethasone, and 1.7 µM insulin (MDI). After 2 days, culture medium was changed to DMEM supplemented only with 10% FBS. As it is well-documented that this adipocyte cell line synchronously reenters the cell cycle for 1–2 rounds of cell division prior to the onset of adipocyte gene expression [18], [26], total RNA was harvested over time following MDI for a period of 24 hrs that has been shown to encompass one cell cycle [19], [20]. For differentiation determinations, total RNA was harvested from preadipocytes induced to differentiate at 2 day intervals over 10 days which is sufficient time for conversion of density-arrested preadipocytes to fully mature adipocytes [25], [26].

### Real-time RT-PCR

Total RNA was extracted and genomic DNA contamination was removed using the RNeasy Plus Mini Kit (Qiagen) following the manufacturer's protocol and total RNA was quantified with a Nanodrop ND-1000 spectrophotometer. Total RNA (2 µg) was reverse-transcribed to cDNA in a 20 µl reaction volume using a high capacity cDNA reverse transcription kit (Applied Biosystems). The reverse transcription (RT) master mix containing RT buffer, dNTP mix, RT random primers, RNase inhibitor (1.0 U/µl), and MultiScribe RT was added to 2 µg RNA and RNase-free water. Reverse transcription reaction conditions followed the protocol (hold at 25°C for 10 minutes, hold at 37°C for 120 minutes, hold at 85°C for 5 seconds, followed by 4°C indefinitely/RT complete) and utilized the Gene Amp PCR System 9700 thermal cycler (Applied Biosystems) for cDNA synthesis.

PCR amplification was run utilizing the 7500 fast system (Applied Biosystems) that consisted of enzyme activation at 95°C for 20 seconds, followed by 40 cycles of denaturation at 95°C for 3 seconds combined with annealing/extension at 60°C for 30 seconds. All data were analyzed with the ABI 7500 real time PCR system (Applied Biosystems). TaqMan primer probes (Table 1) were purchased from Applied Biosystems. Data were recorded and analyzed with Sequence Detector Software (Applied Biosystems) and graphs visualized with SigmaPlot software. All data are representative of experiments performed at least three times in duplicate. Data are mean ± standard error of the mean (SEM). Relative differences between treated and untreated control samples were analyzed by Student's t-test where a p-value of <0.05 was considered statistically significant.

Validation of endogenous controls was measured as the difference in threshold cycle number (ΔC_T_) between treatment and control and converted to fold changes as previously described [16]. Utilizing the ΔC_T_ method, a one cycle difference correlates to a 2-fold increase or decrease of mRNA. Normalization of target gene to reference gene used the 2^−ΔΔC^_T_ method, where ΔΔC_T_  =  (C_T,target_ – C_T,reference_) _treated sample_ – (C_T,target_ – C_T,reference_) _untreated sample_.
